# Bilateral Ovarian Vein Thrombosis in a Non-Pregnant Woman: A Case Report

**DOI:** 10.7759/cureus.112028

**Published:** 2026-07-04

**Authors:** Khaled M Alghamdi, Mohammed Alnahhas, Adel Alghamdi, Abdulaziz H Basha Ahmed, Ammar Mohammad

**Affiliations:** 1 Internal Medicine, Alnoor Specialist Hospital, Makkah, SAU; 2 Diabetes and Endocrinology Center, Diabetes Center of Hera General Hospital, Makkah, SAU; 3 Adult Hematology, King Faisal Specialist Hospital and Research Centre, Jeddah, SAU

**Keywords:** anticoagulation therapy, apixaban, bilateral ovarian vein thrombosis, fever of unknown origin, ovarian vein thrombosis, venous thromboembolism

## Abstract

Ovarian vein thrombosis (OVT) is a rare vascular disorder most commonly associated with pregnancy and the puerperium; however, it may also occur in non-pregnant women, in whom diagnosis can be challenging because of its rarity and nonspecific clinical presentation. We report the case of a 37-year-old non-pregnant woman with no significant past medical history who presented with a one-month history of undocumented fever associated with right lower quadrant abdominal pain and constitutional symptoms, including weight loss, night sweats, and fatigue. She had delivered nine months before presentation, well beyond the recognized high-risk postpartum period, and denied oral contraceptive use. Laboratory investigations were largely unremarkable, except for a markedly elevated erythrocyte sedimentation rate. Contrast-enhanced computed tomography of the abdomen and pelvis demonstrated bilateral OVT. The patient was admitted with a working diagnosis of fever of unknown origin and was started on therapeutic anticoagulation with low-molecular-weight heparin, followed by a transition to apixaban. Her condition improved clinically, and a structured follow-up plan was arranged. Bilateral OVT in the absence of pregnancy is particularly uncommon and may be overlooked because of the lack of specific clinical and laboratory findings, making imaging essential for diagnosis. Early initiation of anticoagulation is important to reduce the risk of potentially life-threatening complications. Although direct oral anticoagulants are increasingly used in the management of venous thromboembolism, evidence supporting their use specifically in non-pregnancy-related OVT remains limited. This case highlights bilateral OVT as a rare but important cause of prolonged fever and abdominal pain in non-pregnant women and emphasizes the need for early recognition to avoid diagnostic delay and ensure timely management.

## Introduction

Ovarian vein thrombosis (OVT) is a rare but potentially serious puerperal complication [1]. First documented in 1956, it has since been reported mainly in case reports and small observational series [2]. Common clinical features include fever, lower abdominal or flank pain, and a palpable abdominal mass [3]. OVT may be associated with gynecologic malignancies, pelvic surgical procedures, pelvic inflammatory disease (PID), and underlying hypercoagulable conditions [4]. Prompt diagnosis and management are crucial to avoid severe complications such as thrombus extension to the inferior vena cava (IVC), pulmonary embolism (PE), and sepsis [5]. OVT is typically diagnosed through imaging modalities rather than clinical evaluation or surgical exploration. Diagnostic tools may include ultrasonography, computed tomography (CT), or magnetic resonance imaging (MRI) [5].

OVT most commonly occurs during pregnancy and the puerperium. The pathophysiology of OVT is best explained by Virchow’s triad, which consists of venous stasis, endothelial injury, and a hypercoagulable state. Although these three mechanisms contribute to thrombus formation in all patients, the underlying factors differ according to the clinical setting [3].

In postpartum ovarian vein thrombosis (POVT), Virchow’s triad is well established, as pregnancy and the puerperium naturally promote venous stasis, endothelial injury, and a hypercoagulable state. Consequently, the pathogenesis of OVT is well understood in this population. In contrast, OVT in non-pregnant individuals is much less common and is typically associated with systemic prothrombotic conditions or local pathological processes, such as malignancy, PID, pelvic surgery, or inherited and acquired thrombophilias. These conditions contribute to one or more components of Virchow’s triad, ultimately leading to thrombus formation [3,5].

OVT most commonly affects the right ovarian vein (up to 90% of cases) because of anatomical and physiological factors, including its direct drainage into the IVC, the acute angle of venous insertion, and retrograde venous blood flow [5].

The differential diagnosis is broad, necessitating a high index of suspicion. In a retrospective review by Kominiarek and Hibbard, most patients were initially misdiagnosed with pyelonephritis or appendicitis, leading to delays in appropriate treatment [6].

Anticoagulation is the cornerstone of treatment for OVT. In hospitalized or high-risk patients who may require rapid reversal of anticoagulation, unfractionated heparin (UFH) may be more appropriate [3]. Once stabilized, patients are typically transitioned to oral anticoagulants such as warfarin or direct oral anticoagulants (DOACs). While DOACs have demonstrated non-inferiority to warfarin in several large phase III trials for the treatment of venous thromboembolism (VTE) in the general population [7-10], specific data on their efficacy in OVT are lacking, and no direct comparative studies exist for this indication [11-13].

## Case presentation

A 37-year-old female patient with no significant past medical history presented to the emergency department with a one-month history of undocumented fever not responding to paracetamol. The fever was associated with right lower quadrant abdominal pain that began concurrently, along with constitutional symptoms including dry cough, unintentional weight loss, loss of appetite, drenching night sweats, and fatigue. The patient also reported intermittent dysuria and intermittent antibiotic use during the same period.

She denied the use of oral contraceptive pills (last use was two years ago), had no history of thromboembolic events, and reported five full-term deliveries, with the last occurring nine months before presentation. There was no history of recurrent pregnancy loss. Her family history was notable for deep vein thrombosis and PE in her mother during hospitalization for severe COVID-19. Past surgical history included an uncomplicated cholecystectomy performed four years ago. She reported no known drug allergies, and her social history was unremarkable.

On presentation, the patient appeared well and was hemodynamically stable, with a height of 165 cm, a weight of 63.7 kg, and a body mass index (BMI) of 23.4 kg/m².

The patient underwent a comprehensive panel of investigations, including hematological, biochemical, infectious, coagulation, metabolic, and microbiological studies. Overall, the results were within normal limits, with the exception of a markedly elevated erythrocyte sedimentation rate (ESR) of 86 mm/hr (Tables 1-5).

**Table 1:** Complete blood count and related tests WBC, white blood cell count; RBC, red blood cell count; RPI, reticulocyte production index; ESR, erythrocyte sedimentation rate; CRP, C-reactive protein

Test	Reference Range	Result
WBC	4.0–11.0 x10^9^/L	7.8
RBC	4.0–5.2 x10^12^/L (F)	4.7
Hemoglobin	12.0–15.5 g/dL (F)	13.4
Hematocrit	34.9–44.5% (F)	40.1
Platelets	150–450 x10^9^/L	265
Peripheral smear	Normal morphology	Normal
Reticulocyte count	0.5–2.5%	1.2%
RPI	≥2	2.0
ESR	0–20 mm/hr	86
CRP	0–5 mg/L	3.0
Procalcitonin	<0.05 ng/mL	0.02
Lactate	0.5–2.2 mmol/L	1.1

**Table 2:** Electrolytes and renal/liver function tests Na, sodium; K, potassium; BUN, blood urea nitrogen; AST, aspartate aminotransferase; ALT, alanine aminotransferase; LDH, lactate dehydrogenase

Test	Reference Range	Result
Na	135–145 mmol/L	139
K	3.5–5.0 mmol/L	4.1
BUN	7–20 mg/dL	14
Creatinine	0.6–1.2 mg/dL	0.8
Magnesium	1.7–2.2 mg/dL	2.0
Phosphate	2.5–4.5 mg/dL	3.5
Calcium	8.5–10.5 mg/dL	9.2
Albumin	3.5–5.0 g/dL	4.1
AST	10–40 U/L	26
ALT	7–56 U/L	30
Alkaline phosphatase	40–129 U/L	96
Total bilirubin	0.1–1.2 mg/dL	0.7
Direct bilirubin	0.0–0.3 mg/dL	0.2
LDH	140–280 U/L	210
Amylase	30–110 U/L	65
Lipase	0–160 U/L	75

**Table 3:** Coagulation profile INR, international normalized ratio; PT, prothrombin time; PTT, partial thromboplastin time

Test	Reference Range	Result
INR	0.8–1.2	1.0
PT	11–13.5 sec	12.6
PTT	25–35 sec	31
D-dimer	<0.5 µg/mL	0.3

**Table 4:** Metabolic and cardiac markers TC, total cholesterol; LDL, low-density lipoprotein; HDL, high-density lipoprotein; TG, triglycerides; HbA1c, hemoglobin A1c; BNP, B-type natriuretic peptide

Test	Reference Range	Result
Lipid profile	TC<200, LDL<100, HDL>40, TG<150 mg/dL	TC 175, LDL 90, HDL 52, TG 130
HbA1c	<5.7%	5.5%
Vitamin D	30–100 ng/mL	48
Blood glucose (fasting)	70–99 mg/dL	95
Cardiac enzymes (Troponin)	<0.04 ng/mL	<0.01
BNP	<100 pg/mL	68

**Table 5:** Microbiology

Test	Reference Range	Result
Blood culture (aerobic)	No growth	No growth
Blood culture (anaerobic)	No growth	No growth
Sputum culture	No growth	No growth
Urine culture	No growth	No growth
Urinalysis	Normal	Normal

A contrast-enhanced CT of the abdomen and pelvis demonstrated bilateral OVT (Figures 1, 2). She was admitted with an initial diagnosis of fever of unknown origin and was started on enoxaparin on October 5.

**Figure 1:**
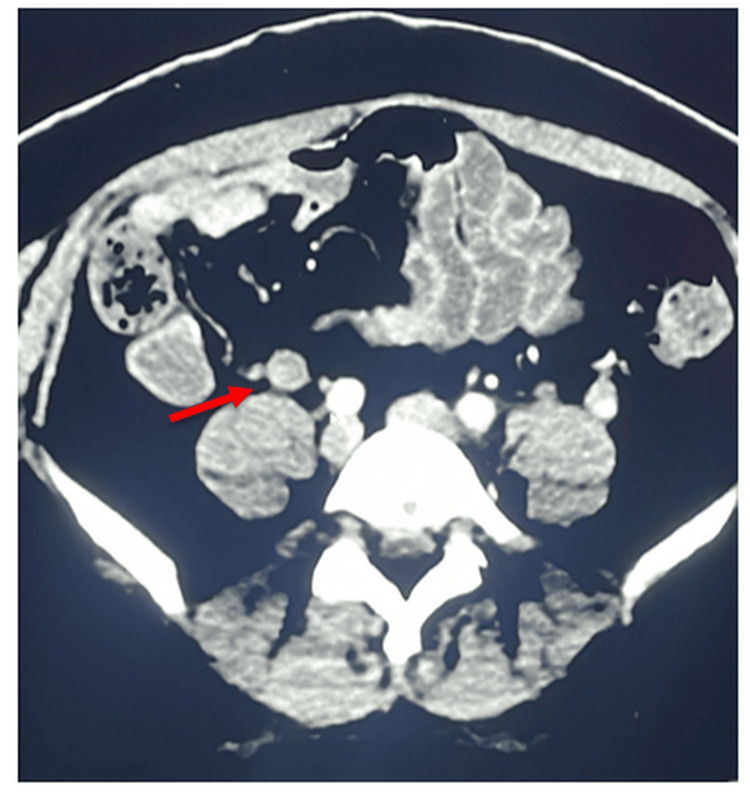
A contrast-enhanced CT of the abdomen and pelvis demonstrating right ovarian vein thrombosis (red arrow)

**Figure 2:**
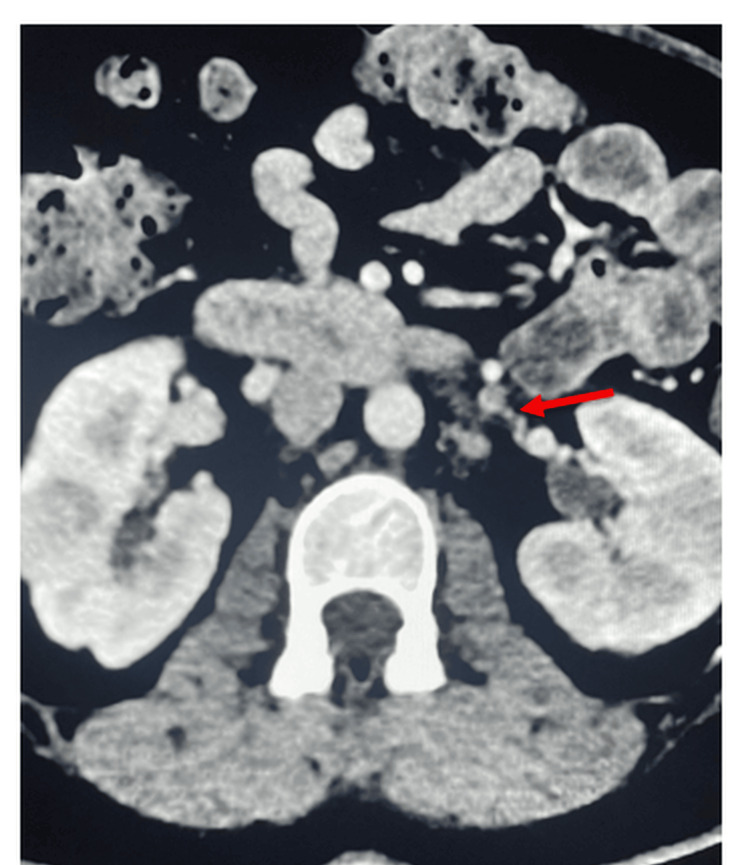
A contrast-enhanced CT of the abdomen and pelvis demonstrating left ovarian vein thrombosis (red arrow)

Given the diagnosis, the patient was planned for discharge with anticoagulation therapy. The discharge regimen included a transition to apixaban: 10 mg twice daily for three days (to complete seven days of full anticoagulation), followed by 5 mg twice daily for three months. The first dose of apixaban was scheduled 12 hours after the last dose of enoxaparin. Follow-up plans included repeating the D-dimer, outpatient evaluation with hematology including a complete blood count (CBC) and coagulation profile, and further thrombophilia workup in the outpatient setting.

Additional recommendations included initiating secondary prophylaxis with apixaban 2.5 mg twice daily after the initial three-month course, rheumatology follow-up, and consideration of CT angiography at the next follow-up visit to assess for recanalization of the ovarian veins.

During the follow-up, the patient underwent further evaluation by the rheumatology team. An extensive thrombophilia and autoimmune workup was completed. Thrombophilia screening, including factor V Leiden mutation, JAK2 mutation, antithrombin, protein C, antiphospholipid antibodies, and lupus anticoagulant, was unremarkable. Autoimmune testing revealed a positive ANA (1:320, homogeneous pattern) with negative anti-dsDNA, anti-Smith antibodies, and normal complement levels. Based on the overall clinical and laboratory findings, a diagnosis of undifferentiated connective tissue disease was considered, and treatment with hydroxychloroquine 200 mg twice daily was initiated. The patient remains on secondary prophylaxis with apixaban 2.5 mg twice daily and continues regular follow-up with the rheumatology team.

## Discussion

OVT is a rare but potentially serious condition that most frequently occurs in the postpartum setting. However, in non-pregnant individuals, OVT presents unique diagnostic and management challenges, often occurring secondary to malignancy, infection, surgery, or inherited hypercoagulable states [1,3,5]. Although the patient had delivered nine months before presentation, she was well beyond the recognized high-risk postpartum period and had no identifiable provoking factors, such as oral contraceptive use, recent surgery, or known thrombophilia, making this an unusual and diagnostically challenging presentation.

The clinical features of OVT are nonspecific and may mimic more common causes of acute abdominal pain such as appendicitis or pyelonephritis, which increase the risk of diagnostic delay [3,6]. This was reflected in our patient’s initial presentation with fever, right lower quadrant abdominal pain, and constitutional symptoms, which led to an initial impression of fever of unknown origin. The diagnosis of bilateral OVT was confirmed by contrast-enhanced CT imaging [5].

The pathophysiology in this case aligns with Virchow’s triad, particularly venous stasis and a possible underlying hypercoagulable state, which was supported by the family history of thromboembolism in the patient’s mother [3]. The right-sided predominance of OVT is well documented due to anatomical factors, but bilateral involvement, as seen in this case, is much rarer and raises the possibility of systemic risk factors or transient triggers such as inflammation or infection [3,5].

OVT in the absence of pregnancy or the postpartum state is uncommon and usually secondary to systemic or local prothrombotic factors, such as malignancy, pelvic surgery, infection, or inherited/acquired thrombophilia [1]. Given this broad differential, a comprehensive diagnostic workup was crucial in this case [1,4].

The evaluation included hematological, biochemical, infectious, coagulation, metabolic, and microbiological studies. With the exception of a markedly elevated ESR (86 mm/hr), all results were within normal limits. The absence of leukocytosis, normal CRP and procalcitonin, and negative microbiological cultures made active infection unlikely. A normal coagulation profile reduced the likelihood of overt systemic coagulopathy, although subtle thrombophilic conditions cannot be excluded without further specialized testing. Normal biochemical and metabolic parameters excluded hepatic, renal, or systemic metabolic dysfunction as underlying contributors.

This pattern of largely unremarkable laboratory findings highlights an important feature of non-POVT: the condition often lacks supportive laboratory evidence and cannot be diagnosed on blood tests alone [4]. The elevated ESR, while nonspecific, provided a marker of underlying inflammation or thrombosis. Ultimately, the workup was valuable in excluding alternative diagnoses and systemic precipitants, thereby supporting the need for definitive imaging to establish the diagnosis.

Although D-dimer is widely used in the evaluation of VTE, its diagnostic utility in OVT is less well established, and a normal D-dimer level does not reliably exclude the diagnosis. In our patient, symptoms had been present for approximately one month before diagnosis, which may have contributed to the negative D-dimer result. This case highlights that persistent clinical suspicion, together with appropriate imaging, remains essential when the clinical presentation is suggestive despite unremarkable laboratory findings [14].

Management of OVT typically involves prompt initiation of anticoagulation therapy, which is critical in preventing complications such as IVC extension, PE, or sepsis [6]. In this patient, initial treatment with enoxaparin, a low-molecular-weight heparin (LMWH), was appropriate given its predictable pharmacokinetics and ease of use in the hospital setting. For hospitalized patients in whom rapid reversal may be necessary, UFH remains an alternative; however, in this case, LMWH was clinically effective and well tolerated [3].

Upon stabilization, the patient was appropriately transitioned to apixaban, a DOAC, with an appropriate lead-in dose and duration. Although DOACs have demonstrated non-inferiority to warfarin for VTE in the general population [8-11], data specific to OVT are limited, and no randomized controlled trials have evaluated DOAC efficacy for this indication [7,12,13]. The decision to proceed with apixaban was supported by its established efficacy and safety in the treatment of VTE, the patient's non-lactating status, the absence of contraindications, convenient oral administration, and a planned hematology follow-up to assess thrombophilia and guide long-term management.

This case illustrates the complexity of diagnosing and managing OVT outside the recognized high-risk postpartum period. It highlights the importance of clinical vigilance, imaging-based diagnosis, and individualized anticoagulation strategies based on risk profiles, comorbidities, and patient-specific factors.

## Conclusions

OVT remains a rare but potentially serious vascular condition, particularly when it occurs outside the immediate postpartum period. This case of bilateral OVT in a non-pregnant woman without major identifiable risk factors highlights the importance of maintaining a high index of suspicion in patients presenting with prolonged fever and abdominal pain, as timely imaging is essential for establishing the diagnosis. Early anticoagulation remains the cornerstone of management and may help prevent potentially life-threatening complications. Although DOACs represent a practical therapeutic option, evidence specific to OVT remains limited, and current management is largely extrapolated from broader VTE data. Further prospective studies are needed to define the optimal anticoagulant, treatment duration, and follow-up strategy for this uncommon condition.
